# Increasing the Magnesium Concentration in Various Dialysate Solutions Differentially Modulates Oxidative Stress in a Human Monocyte Cell Line

**DOI:** 10.3390/antiox9040319

**Published:** 2020-04-15

**Authors:** Carmen Vida, Julia Carracedo, Patricia de Sequera, Guillermo Bodega, Rafael Pérez, Matilde Alique, Rafael Ramírez

**Affiliations:** 1 Departamento de Biología de Sistemas, Universidad de Alcalá, Alcalá de Henares, 28871 Madrid, Spain; 2 Departamento Genética, Fisiología y Microbiología (Sección Fisiología), Universidad Complutense de Madrid, 28040 Madrid, Spain; 3 Instituto de Investigación Sanitaria Hospital 12 de Octubre (imas12), 28041 Madrid, Spain; 4 Sección de Nefrología, Hospital Universitario Infanta Leonor, 28031 Madrid, Spain; 5 Departamento de Medicina, Universidad Complutense de Madrid, 2804 Madrid, Spain; 6 Departamento de Biomedicina y Biotecnología, Universidad de Alcalá, Alcalá de Henares, 28805 Madrid, Spain; 7 Instituto Ramón y Cajal de Investigación Sanitaria, (IRYCIS), 28034 Madrid, Spain

**Keywords:** chronic kidney disease, dialysates, lipid damage, magnesium, monocytes, oxidative stress, uremic toxins, reactive oxygen species

## Abstract

Oxidative stress is exacerbated in hemodialysis patients by several factors, including the uremic environment and the use of dialysis fluids (DFs). Since magnesium (Mg) plays a key role in modulating immune function and in reducing oxidative stress, we aimed to evaluate whether increasing the Mg concentration in different DFs could protect against oxidative stress in immunocompetent cells in vitro. Effect of ADF (acetate 3 mM), CDF (citrate 1 mM), and ACDF (citrate 0.8 mM + acetate 0.3 mM) dialysates with Mg at standard (0.5 mM) or higher (1, 1.25, and 2 mM) concentrations were assessed in THP-1 monocyte cultures. Reactive oxygen species (ROS) and malondialdehyde (MDA) levels were quantified under basal and uremic conditions (indoxyl sulfate (IS) treatment). Under uremic conditions, the three DFs with 0.5 mM Mg promoted higher ROS production and lipid damage than the control solution. However, CDF and ACDF induced lower levels of ROS and MDA, compared to that induced by ADF. High Mg concentration (1.25 and/or 2 mM) in CDF and ACDF protected against oxidative stress, indicated by reduced ROS and MDA levels compared to respective DFs with standard concentration of Mg. Increasing Mg concentrations in ADF promoted high ROS production and MDA content. Thus, an increase in Mg content in DFs has differential effects on the oxidative stress in IS-treated THP-1 cells depending on the dialysate used.

## 1. Introduction

Chronic kidney disease (CKD) patients have a high incidence of cardiovascular disease (CVD), which remains the leading cause of morbimortality in hemodialysis (HD) patients [1,2,3]. Several factors are closely associated with the pathophysiology of both CKD and CVD [3]. Among them, non-dialyzable uremic toxins (UTs), such as indoxyl sulfate (IS), may be one of the critical links between impaired renal function and the prevalence of cardiovascular events through altered leukocyte activation and intensified oxidative stress [3,4,5,6,7].

Classically, UTs are divided into three categories: low-molecular-weight water-soluble solutes (<500 Da), middle-molecular-weight solutes (>500 Da), and protein-bound solutes [8]. These uremic toxins, represented by IS, is extremely toxic and difficult to eliminate by conventional dialysis; consequently, high IS levels may trigger oxidative-inflammatory stress, playing a key pathophysiologic role in CKD. In fact, several studies have confirmed that the occurrence of CVD induced by CKD is closely related to the IS accumulation [3]. 

Uremia promotes a state of enhanced oxidative stress, which is the result of pro-oxidant molecules, such as reactive oxygen species (ROS), overwhelming the antioxidant defense mechanisms [9]. An increase in oxidative stress occurs during the dialysis session owing to the loss of antioxidants (e.g., uric acid, etc.) and the activation of leukocytes, which trigger both inflammatory compounds and ROS production, contributing to oxidative damage of biomolecules (e.g., lipid peroxidation, DNA damage, etc.) [3,9,10,11,12].

Clinical and experimental evidence demonstrates that several factors inherent to the dialysis procedure itself, such as dialysis modality, iron administration, biocompatibility, and types of dialyzer membrane and dialysates, can play a key role promoting chronic inflammation and oxidative stress in HD patients [9,10,11,12]. Bio-incompatibility during HD induces leukocyte activation, which results in increased ROS production and release of pro-inflammatory cytokines [13,14]. Dialysis fluids (DFs) per se can induce an increase in the production of pro-inflammatory molecules and oxidizing compounds (e.g., ROS) [13,14,15,16]. However, this effect depends on the composition of the DFs, which is critical to optimize removal of UTs, and preserving fluid, electrolyte, and acid-base balance [17]. DFs are electrolyte solutions prepared by mixing purified water with both an acid and a base concentrate. To avoid the precipitation of both calcium and magnesium carbonates that takes place in the DFs when adding bicarbonate, it is necessary to add an acid, like acetic acid (usually at 3–10 mM concentrations) [18,19]. However, after a standard HD session, its plasma level becomes 30 to 40 times higher than physiologic concentration, promoting several side effects (e.g., endothelial dysfunction, etc.). Moreover, acetate also induces leukocytes activation, promoting increased ROS production and release of inflammatory compounds [13,14,15,16]. Recently, citric acid has been proposed as a valuable alternative to acetate due to its anticoagulant, anti-inflammatory, and antioxidant properties [20]. Indeed, citrate chelates multivalent cations (e.g., calcium, iron, magnesium, etc.) and reduces complement and leukocytes activation, improving dialysis efficiency [19,20].

Magnesium (Mg) is very important for many basic physiological functions in the human body [21]. Epidemiological studies have demonstrated an association between hypomagnesemia (low serum Mg concentration) and CVD-associated mortality in CKD patients [21,22,23,24]. Moreover, exacerbated oxidative-inflammatory stress has been associated with hypomagnesemia in HD patients [12,24]. Thus, it has been proposed that increasing the Mg concentration in DFs may be a useful strategy to prevent CVD associated oxidative stress in HD patients [12,21,22,24,25,26,27,28]. Mg concentration in DFs is one of the significant determinants of Mg serum balance in HD patients [25]. However, the optimal Mg concentration in the dialysate has not yet been determined [25,29]. A recent multicenter, randomized, and prospective study suggests the use of citrate dialysates with a higher Mg concentration than the usual (0.5 mM) [30]. Since citrate may chelate Mg ions, high Mg levels in dialysate might be beneficial and needed when using ≥ 1 mM citrate concentrates [16,19,30]. By contrast, some studies have revealed detrimental effects on the addition of high Mg doses in acetate dialysates, such as alterations to bone metabolism and parathyroid gland function, promoting calcification and cardiovascular events [28,29,30,31,32]. More studies are necessary to understand the beneficial or harmful effects of the increase of Mg concentrations in dialysates in the context of renal replacement therapy [24].

Based on the above considerations, this study aims to evaluate in vitro the direct effects of higher Mg concentrations in different DFs (acetate, citrate, or both) on modulating oxidative stress and damage in IS-treated human monocytes as well as identify potential differences between the use of citrate and acetate in DFs under uremia toxicity. 

## 2. Materials and Methods

### 2.1. Hemodialysis Fluids

Three types of DFs were used:

ADF: SoftPac^®^ (Baxter, Madrid, Spain) containing 3 mM acetate and no citrate; 

ACDF: Citrasate^®^ (Nipro, Lérida, Spain) containing 0.3 mM acetate and 0.8 mM citrate;

CDF: SelectBag Citrate^®^ (Baxter, Madrid, Spain) containing 1 mM citrate and no acetate.

The compositions of the DFs are shown in Table 1. Hank’s medium, with basal Mg concentration similar to that in DFs, was used as a control solution.

### 2.2. Culture of Human Monocytes (THP-1)

Human THP-1 cells, a pro-monocytic cell line (Sigma-Aldrich, Cat#88081201, San Louis, MO, USA), were cultured in RPMI-1640 medium with 1% penicillin-streptomycin, both supplied by Lonza (Basel, Switzerland) and supplemented with 10% fetal bovine serum (FBS, Sigma-Aldrich, San Louis, MO, USA). THP-1 cells were cultured at a high density (1.0 × 10^6^/mL), with media being refreshed every 4–5 days and were maintained at 5% CO_2_, 37 °C. For the different experiments, THP-1 cells were treated with IS 256 µg/mL diluted in PBS.

According to a previous model of endothelial damage using different doses of IS to mediate oxidative stress in endothelial cells, the dose of IS 256 μg/mL was optimal to induce cell activation without producing toxicity and cell death [33]. After performing a dose-response study to analyze the cellular viability, we selected the dose of IS at 256 μg/mL to induce cell activation in THP-1 monocytes.

### 2.3. Quantification of Reactive Oxygen Species Levels

The intracellular ROS generation in THP-1 monocytes was measured using a method described by Wang and Joseph [34] based on the fluorescence emitted by 2′,7′-dichlorodihydrofluorescein (DCF-DA, Sigma-Aldrich, San Louis, MO, USA) when oxidized in the cytoplasm by ROS to 2′,7′-dichlorofluorescein. Briefly, aliquots of 100 µL of THP-1 suspension adjusted to 2 × 10^6^ cells/mL in RPMI-1640 medium were plated into 96-well plates (Nunclon Delta, Thermo Fisher Scientific, Waltham, Massachusetts, USA). The cells were (pre)treated with different Mg concentrations: 1, 1.25, and 2 mM for 30 min (37 °C) and then incubated for 30 min (37 °C) with IS (256 µg/mL) along with 100 µL of various DFs or control solution. After incubation with IS, cells were incubated for 30 min (37 °C) with DCF-DA (1 mM). Fluorescence was recorded with excitation/emission at 485–535 nm at different times (30, 60, 90, 120, 150, and 180 min) after the addition of DCF-DA. The results were expressed as “relative units of fluorescence” (URF) and as a percentage (%) of corresponding basal value without IS. 

### 2.4. Lipid Peroxidation Assay

Quantification of malondialdehyde (MDA) levels was performed using the commercial ‘‘Lipid peroxidation (MDA) Assay Kit’’ (Biovision, Milpitas, CA, USA), which measures the reaction of MDA with thiobarbituric acid (TBA) and the MDA–TBA adduct formation. The assay was evaluated in aliquots of frozen THP-1 monocytes (3 × 10^6^ cells), which were previously cultivated in ADF, CADF, CDF, and the control solution with different Mg concentrations (0.5, 1, 1.25, and 2 mM), and then incubated for 4 h (37 °C and 5% CO_2_) with or without IS (256 μg/mL). Cells were resuspended in MDA lysis buffer containing butylated hydroxytoluene (BHT; 0.1 mM), sonicated, and centrifuged at 13,000 × *g* for 10 min. Supernatants were collected, mixed with TBA (Sigma-Aldrich, San Louis, MO, USA), and incubated in a water bath at 95 °C for 60 min. Then, samples were cooled in ice for 10 min and mixed with n-butanol (Sigma-Aldrich, San Louis, MO, USA) to create an organic phase in which the MDA molecules were to be placed. Samples were centrifuged at 13,000 × *g* for 10 min, and the supernatants were collected and dispensed into a 96-well microplate for spectrophotometric measurement at 532 nm. The protein contents were evaluated following the bicinchoninic acid protein (BCA) assay kit protocol (Sigma-Aldrich, San Louis, MO, USA) according to the manufacturer’s protocols. Serum albumin (Sigma-Aldrich, San Louis, MO, USA) was used as a standard. Results are expressed as nanomoles of MDA per milligram of protein.

### 2.5. Cell Viability Assay

The viability of THP-1 monocytes was assessed by the 3-(4,5-dimethylthiazol-2-yl)-2,5-diphenyltetrazolium bromide (MTT) assay kit (Sigma-Aldrich, San Louis, MO, USA) according to the manufacturer’s protocols. Cells were seeded in 96-well plates (0.2 × 10^6^ cells/well) and stained with MTT solution (0.5 mg/mL) for 4 h at 37 °C and 5% CO_2_. The medium was removed, and formazan solubilized by adding 100 μL/well of dimethyl-sulfoxide (Sigma-Aldrich, San Louis, MO, USA). The optical density (OD) was measured at 570 nm. Results are expressed as percentages (%) relative to the results obtained with control cells.

### 2.6. Statistical Analysis

Statistical analyses were performed with the SPSS 21.0 statistical package (SPSS Inc., Chicago, IL, USA). All data are expressed as mean ± standard deviation (SD). All tests were two-tailed, with a significance level of α = 0.05. The normality of the samples and the homogeneity of the variances were checked by the Kolmogorov-Smirnov and Levene tests, respectively. Differences between groups were studied by Student’s *t*-test analysis.

## 3. Results

### 3.1. ROS Production in THP-1 Cells Induced by IS Treatment in Different Dialysis Fluids

**Figure 1:**
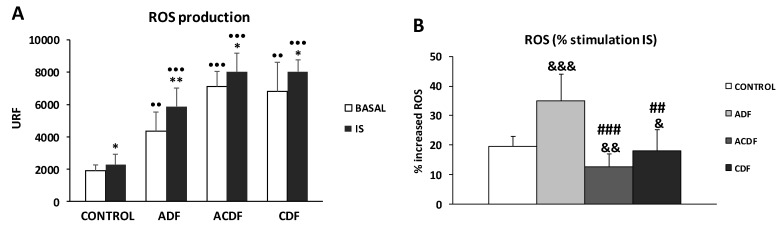
Intracellular reactive oxygen species (ROS) production under basal conditions and in response to indoxyl sulfate (IS, 256 µg/mL), in human THP-1 monocytes cultured in control solution (Hank’s medium) or in different dialysis fluids (ADF, 3 mM acetate; ACDF, 0.8 citrate + 0.3 mM acetate; CDF, 1 mM citrate). (**A**) ROS levels (URF) and (**B**) percentage (%) increase in ROS production by THP-1 cells after 180 min of stimulation with IS. Each column represents the mean ± SD of 5–8 independent experiments. * *p* < 0.05 and ** *p* < 0.01 vs. the value in basal conditions. ^••^ *p* < 0.01, and ^•••^ *p* < 0.001 vs. the value in the control solution. ^##^ *p* < 0.01 and ^###^ *p* < 0.001 vs. the value ADF. ^&^ *p* < 0.05, ^&&^ *p* < 0.01, and ^&&&^ *p* < 0.001 vs. the value of % of ROS in the control solution.

THP-1 monocytes were grown in the presence of three different DFs (ADF, ACDF, and CDF) with similar standard Mg concentrations (0.5 mM). Intracellular ROS production was measured under basal and IS-treated conditions (Figure 1 and Figure S1).

Under basal conditions, we observed that ADF, ACDF, and CDF induced significantly higher ROS production (*p* < 0.01 in ADF and CDF; *p* < 0.001 in ACDF) compared to the control solution (Figure 1A). Interestingly, under uremic conditions, monocytes cultured with ADF, ACDF, and CDF produced a higher ROS production (*p* < 0.001) than those observed in the control solution (Figure 1A). It is important to note that, after IS-stimulation, we found that the control as well as DFs (ADF, ACDF, and CDF) stimulated THP-1 cells have enhanced release of ROS, which are 19%, 36%, 13%, and 17% higher, respectively, compared to corresponding baseline levels (Figure 1B). Furthermore, although the basal ROS levels in ADF were lower than those observed in ACDF and CDF (ROS: 4373 vs. 7115 and 6822 URF; Figure 1A), analysis of the % of ROS production in response to IS a marked increase in % ROS was observed in monocytes incubated with ADF (*p* < 0.001) in relation to control solution (Figure 1B). On the other hand, ACDF and CDF (*p* < 0.01 and *p* < 0.05, respectively) induced lower % ROS production in response to IS compared to the levels in control solution, as well as in comparison to ADF (*p* < 0.001 and *p* < 0.01 in ACDF and CDF vs. ADF; Figure 1B). 

### 3.2. High Concentration of Mg Attenuates ROS Production in IS-Stimulated THP-1 Cells

**Figure 2:**
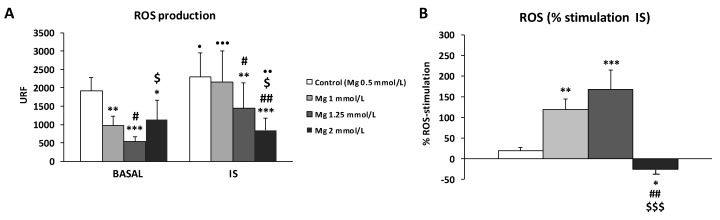
Intracellular reactive oxygen species (ROS) production under basal conditions and in response to indoxyl sulfate (IS) treatment (256 µg/mL) in human THP-1 monocytes cultured in control solution (Hank’s medium) with the standard Mg (0.5 mM; control) or with high Mg concentrations (1, 1.25, and 2 mM). (**A**) ROS levels (URF) and (**B**) percentage (%) increases in ROS production after 180 min of stimulation with IS. Each column represents the mean ± SD of 5–8 independent experiments. * *p* < 0.05, ** *p* < 0.01, and *** *p* < 0.001 vs. the corresponding 0.5 mM Mg (control). ^#^ *p* < 0.05 and ^##^ *p* < 0.01 vs. the corresponding 1 mM Mg. ^$^ *p* < 0.05 and ^$$$^ *p* < 0.001 vs. the corresponding 1.25 mM Mg. ^•^ *p* < 0.05, ^••^ *p* < 0.01, and ^•••^ *p* < 0.001 vs. the corresponding basal conditions.

In this study, we investigated whether the addition of higher concentrations of Mg in a control solution could affect ROS production in THP-1 monocytes cultured under basal or IS-stimulated conditions (Figure 2 and Figure S2). Our results show that an increase in Mg (1, 1.25, and 2 mM) resulted in a significant reduction in basal ROS production, which was lowest with 1.25 mM Mg (*p* < 0.001) (Figure 2A). Similar results were also observed with IS treatment group, where dose-dependent reduction in ROS levels were observed with 1.25- and 2-mM Mg (*p* < 0.01 and *p* < 0.001, respectively) compared to the control (standard Mg, 0.5 mM) (Figure 2A). Interestingly, although ROS production in the presence of 0.5, 1, 1.25, and 2 mM Mg was higher with the IS treatment than at the baseline conditions when we analyzed the percentage increase of ROS production in response to IS, we observed that it decreased very markedly in the presence of 2 mM Mg in comparison to the control (0.5 mM Mg) (*p* < 0.05; Figure 2B). 

### 3.3. Effect of High Mg Concentration on Different Dialysate-Induced ROS Production in IS-Stimulated THP-1 Cells

**Figure 3:**
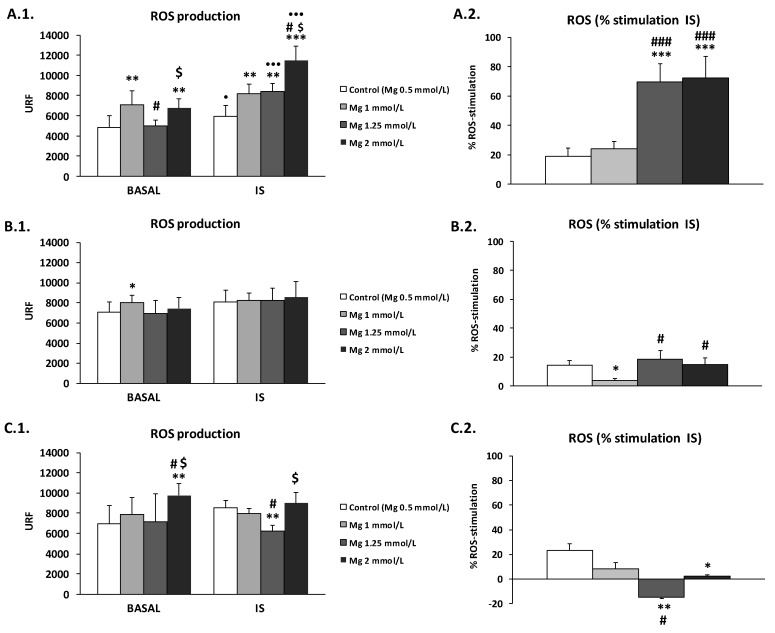
Intracellular reactive oxygen species (ROS) production under basal conditions and in response to indoxyl sulfate (IS) treatment (256 µg/mL) in human THP-1 monocytes cultured in (**A**) ADF (acetate 3 mM), (**B**) ACDF (citrate 0.3 mM + acetate 0.8 mM), and (**C**) CDF (citrate 1 mM) dialysates with standard (0.5 mM, control) or with high Mg concentrations (1, 1.25, and 2 mM). (**1**) ROS levels (URF) and (**2**) percentage (%) of increases in ROS production after 180 min of stimulation with IS. Each column represents the mean ± SD of 5–8 independent experiments. * *p* < 0.05, ** *p* < 0.01, and *** *p* < 0.001 vs. the corresponding 0.5 mM Mg (control). ^#^ *p* < 0.05 and ^###^ *p* < 0.001 vs. the corresponding 1 mM Mg. ^$^ *p* < 0.05 vs. the corresponding 1.25 mM Mg. ^•^ *p* < 0.05 and ^•••^ *p* < 0.001 vs. the corresponding basal conditions.

In the next experiment, we used high doses of Mg (1, 1.25, and 2 mM) in ADF, ACDF, and CDF to test their effect on ROS production by THP-1 cells (Figure 3 and Figure S3). Interestingly, the effect was different depending on the type of dialysate used.

Under basal conditions, Mg in ADF induced a significant increase in ROS production (1 mM Mg, *p* < 0.01; 2 mM Mg, *p* < 0.001; Figure 3A.1). Similar results were also observed in ACDF and CDF, where increased ROS levels were observed with 1.25 mM Mg (*p* < 0.05; Figure 3B.1) and 2 mM Mg (*p* < 0.01; Figure 3C.1), respectively, in relation with the levels observed with 0.5 mM Mg. 

Under uremic conditions, the effects on ROS production were different depending on the type of DF used. An increase in the concentration of Mg in ADF amplified ROS production in a dose-dependent manner (Figure 3A.1), the level being highest with 2 mM Mg (*p* < 0.001). On the contrary, in the case of CDF (Figure 3C.1), 1.25 mM Mg markedly reduced the ROS level compared to that observed with 0.5 mM Mg. On the other hand, similar levels of ROS were observed in ACDF with 0.5, 1, 1.25, and 2 mM Mg (Figure 3B.1). Interestingly, when we analyzed the percentage increase of ROS production in response to IS, we observed that high doses of Mg in CDF dose-dependently reduced the percentage of ROS (*p* < 0.01 and *p* < 0.05 with 1.25 and 2 mM Mg, respectively; Figure 3C.2) in comparison to the standard 0.5 mM Mg, or to its baseline level (*p* < 0.01 with 1.25 mM Mg; Figure 3C.1). Similar results were observed in the case of ACDF; however, the percentage of ROS production in response to IS was reduced only with 1 mM Mg (*p* < 0.05; Figure 3B.2). On the other hand, high Mg concentration in ADF rendered the cells more sensitive to IS, inducing a significantly higher level of ROS production (*p* < 0.001 with 1.25 and 2 mM Mg) compared to the control (Figure 3A.2). 

### 3.4. Effect of High Mg Concentration on Different Dialysate-Induced Lipid Oxidative Damage in IS-Stimulated THP-1 Cells

**Figure 4:**
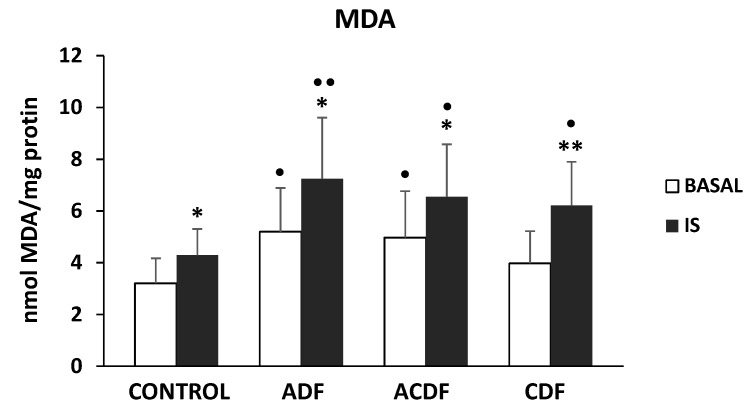
Intracellular malondialdehyde (MDA) levels under basal conditions and in response to indoxyl sulfate (IS; 256 µg/mL) in human THP-1 monocytes cultured in control solution (Hank’s medium) or in ADF (acetate 3 mM), ACDF (citrate 0.3 mM + acetate 0.8 mM), and CDF (citrate 1 mM) dialysates. Each column represents the mean ± SD of 5–8 independent experiments. * *p* < 0.05 and ** *p* < 0.01 vs. the value in the basal conditions. ^•^ *p* < 0.05 and ^••^ *p* < 0.01 vs. the corresponding value in the control solution.

**Figure 5:**
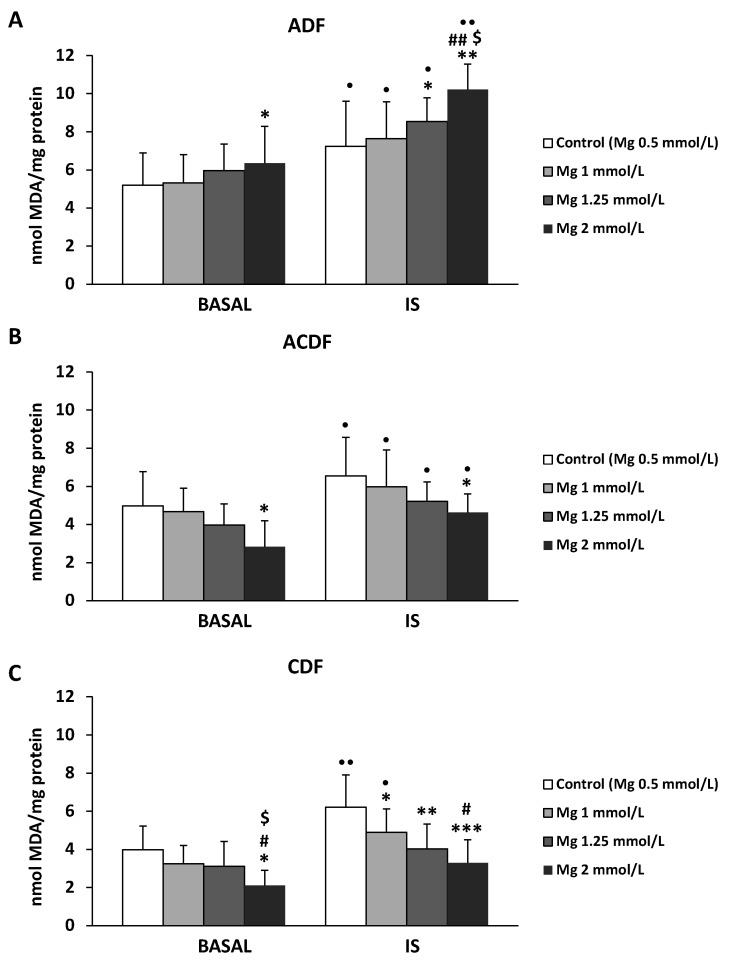
Intracellular malondialdehyde (MDA) levels under basal conditions and in response to indoxyl sulfate treatment (IS; 256 µg/mL) in human THP-1 monocytes cultured in (**A**) ADF (acetate 3 mM), (**B**) ACDF (citrate 0.3 mM + acetate 0.8 mM), and (**C**) CDF (citrate 1 mM) dialysates with standard Mg (0.5 mM, control) or with high Mg concentrations (1, 1.25, and 2 mM). Each column represents the mean ± SD of 5–8 independent experiments. * *p* < 0.05, ** *p* < 0.01, and *** *p* < 0.001 vs. the corresponding 0.5 mM Mg (control). ^#^ *p* < 0.05 and ^##^ *p* < 0.001 vs. the corresponding 1 mM Mg. ^$^ *p* < 0.05 vs. the corresponding 1.25 mM Mg. ^•^ *p* < 0.05 and ^••^ *p* < 0.01 vs. the corresponding basal conditions.

Since high ROS levels could promote oxidative damage, we also investigated whether the supplementation of ADF, ACDF, and CDF with high doses of Mg (1, 1.25, and 2 mM) could prevent oxidative lipid damage in THP-1 monocytes cultured under basal or IS-stimulated conditions. We observed that monocytes cultured in ADF, ACDF, and CDF with the standard 0.5 mM Mg presented higher MDA levels than those cultured in a control solution with the same Mg concentration (Figure 4). These results were observed under basal conditions (*p* < 0.05 in ADF and ACDF), and more significantly with IS treatment (*p* < 0.05 in ACDF and CDF; *p* < 0.01 in ADF). Furthermore, in the three DFs analyzed, IS treatment induced significantly higher levels of MDA than those observed under basal conditions (*p* < 0.05 in ADF and ACDF; *p* < 0.01 in CDF) (Figure 4 and Figure 5). Nevertheless, the effect of the addition of Mg to the DFs was different depending on the type of dialysate used (Figure 5). Interestingly, ACDF showed a very different behavior compared to both CDF and ACDF. The MDA levels increased after incubation with ADF containing 1.25 or 2 mM Mg in (*p* < 0.05 and *p* < 0.01, respectively; Figure 5A), especially after treatment with IS. By contrast, IS-treated monocytes cultured with CDF containing high Mg (1, 1.25, and 2 mM) showed significantly lower MDA levels (*p* < 0.05, *p* < 0.01, and *p* < 0.001, respectively; Figure 5C) than those observed with the standard 0.5 mM Mg. Similar results were observed in monocytes cultured with ACDF, where 2 mM Mg caused a marked reduction in MDA levels (*p* < 0.05; Figure 5B) under basal and uremic conditions. 

### 3.5. Effect of High Mg Containing Dialysates on the Viability of IS-Treated THP-1 Monocytes

The DFs (ADF, ACDF, and CDF) with standard concentration of Mg (0.5 mM) did not significantly affect the viability of THP-1 cells, as measured by MTT assay (cell viabilities were 98% ± 5%, 93% ± 8%, 95% ± 6%, and 96% ± 11% in the control solution, ADF, ACDF, and CDF, respectively. The cell viabilities after exposure to 1, 1.25, and 2 mM Mg concentrations were: 96% ± 6%, 95% ± 10%, and 97% ± 9% in the control solution; 94% ± 7%, 90% ± 11%, and 88% ± 8% in ADF; 93% ± 9%, 92% ± 11%, and 91% ± 8% in ACDF; and 94% ± 9%, 93% ± 4%, and 94% ± 9% in CDF, respectively. A significant difference was observed between IS-treated monocytes cultured in ADF with 0.5 mM and 2 mM Mg (*p* < 0.05).

## 4. Discussion

Increased oxidative stress and reduced serum Mg levels in CKD patients have been associated with uremia and several factors associated with the HD procedure, such as composition of DFs. [9,11,16]. Owing to the positive impact of citrate containing fluids on the hemodynamic parameters and redox status of HD patients, it has recently been proposed to use citrate, instead of the less physiologic acetate, in DFs to improve the outcome of HD process in clinical practice [16,35]. Moreover, Mg deficiency is involved in the development and maintenance of an elevated oxidative stress status, contributing to CVD in HD patients [11,24,36]. Therefore, the use of DFs with high Mg concentrations could prevent uremia-induced oxidative stress in HD patients [11,12]. In this study, we used an in vitro model to examine how higher concentrations (1, 1.25, and 2 mM) of Mg in different DFs (acetate, citrate, or both) would affect their induction of several oxidative stress parameters in THP-1 monocytes, under basal or uremic conditions. 

To our knowledge, this is the first study that demonstrated that the increase in Mg concentration in DFs has either beneficial or deleterious effects on oxidative stress in monocytes depending on the type of dialysate used. Interestingly, high Mg levels in CDF (citrate) protected against ROS production and oxidative lipid damage in a dose-dependent manner (marked reduction of ROS and MDA levels with 1.25 and 2 mM Mg). By contrast, this protective effect was not observed with ACDF (acetate), while the increase of Mg in ADF promoted high ROS production and lipid peroxidation, especially with 2 mM Mg.

Several studies revealed that IS induce endothelial dysfunction and promotes oxidative-inflammatory stress by inducing ROS production in leukocytes, also contributing to CVD and immune dysfunctions in CKD patients [37,38,39,40,41]. Moreover, it has been described that DFs themselves differentially induce ROS production [16]. In our study, IS-treatment remarkably increased ROS production in monocytes cultured in the three DFs (with standard 0.5 mM Mg). However, an enhancement of IS-induced ROS production was observed with ADF in comparison to the control solution, whereas in both ACDF and CDF the % ROS-stimulation was lower than the control solution and the ADF. This could be because citrate has a direct inhibitory effect on leukocyte activation, reducing pro-inflammatory compounds and ROS production [16,42]. By contrast, the use of ADF has been associated with increased acetatemia, inducing leukocytes activation and, consequently, promoting oxidative-inflammatory stress [16]. Our results agree with previous studies, which revealed that citrate (or acetate-free) dialysates induced lower pro-inflammatory cytokine release and ROS generation than acetate dialysates [16,35,43,44]. Interestingly, citrate compounds also regulate redox signaling in different cells and tissues, probably owing to their antioxidant and anti-inflammatory properties [45]. In fact, citrate increases antioxidant enzymes (e.g., superoxide dismutase, glutathione) and decreases the levels of inflammatory and oxidizing compounds, also suppressing NF-ĸB activation [45]. This regulation of redox signals in cells could explain the observed protective effects of CDF and ACDF on monocytes under uremic oxidative stress.

Low Mg levels are associated with CVD and high mortality in dialysis patients [3,21,24,46]. Serum Mg in HD patients is affected by the dialysate Mg concentration, which is low (0.5 mM) as standard [23,28,46], and may induce a post-dialytic hypomagnesemia and undesirable hemodynamic effects, such as hypotension, arrhythmia, and calcification) [23,30,46,47]. Furthermore, due to anti-inflammatory and antioxidant properties of Mg, hypomagnesemia has also been associated with increased oxidative-inflammatory stress [36,48]. In fact, low Mg transitorily leads to pro-oxidant effects, exacerbating ROS production [36,49]. It is unclear whether Mg deficit can be restored by dietary intake [47]. Therefore, it has been proposed that a slightly elevated Mg concentration in DFs may be effective to prevent CVD associated with oxidative-inflammatory stress in HD patients [46,47]. However, there is still no consensus regarding the optimal dialysate Mg concentration [25]. Our study clearly showed that the addition of Mg (1, 1.25, and 2 mM) in ADF, ACDF, or CDF had different effects modulating oxidative stress in monocytes.

Our data suggested that the increase of Mg concentrations in CDF and ACDF, but not in ADF solution, could have a positive effect on decreasing uremia-associated ROS production. Several in vitro studies have demonstrated that Mg deficiency enhances cytotoxicity, ROS production, and DNA damage in endothelial cells [36,49,50]. Meanwhile, high Mg levels modulate vascular endothelial cells, enhancing both monogenic response to angiogenic factors and proliferation [51]. 

The use of CDFs might affect Mg concentration because citrate may form a complex with Mg, which is dialyzable and increase intradialytic Mg removal [16,27,28,46]. Therefore, serum Mg concentration in HD patients should be measured regularly and adjusted accordingly within normal range [23,46]. Several authors propose the use of a CDF formulation with higher Mg levels than usual (0.5 mM) to solve the aforementioned problem and provide a new strategy to reduce oxidative stress induced by HD therapy [15,26,29]. In a recent retrospective case-control study, HD patients on dialysate with low- or high-Mg (0.5 and 0.75 mM, respectively) were analyzed for survival [32]. During a 3-year follow-up, 20% of the patients died in the high-Mg group vs. 36% in the low-Mg group [32]. These data suggest that the increased Mg concentration in the dialysate could have a beneficial effect on CVD-associated mortality in HD patients [23,28,46]. 

Under uremic conditions, both enhanced oxidative stress and Mg deficiency can modify circulating lipids and lipoproteins, leading to lipid peroxidation [24,36]. An increase in oxidative lipid damage is closely associated with the HD process, mainly through leukocytes activation [9,11]. In fact, lipid peroxidation products, such as MDA, are increased in HD patients, promoting inflammation and cardiovascular dysfunction [9,11,52,53]. Our study showed that IS-treatment remarkably increases the MDA in monocytes cultured in ADF, ACDF, and CDF with standard 0.5 mM Mg. Interestingly, higher concentration of Mg in CDF dose-dependently decreased MDA content in monocytes, especially under uremic conditions. Meanwhile, 1.25- and 2-mM Mg remarkably increased the MDA content in monocytes cultured with ADF. To our knowledge, there are no in vitro studies that have evaluated the effect of increasing Mg concentration in different DFs. A study conducted in Japan showed that HD with CDF decreases glycoxidation and MDA levels compared with conventional ADF [14]. Another study also demonstrated that citrate decreases degranulation of polymorphonuclear leukocytes and platelets and, consequently, reduces oxidative stress and lipid peroxidation during HD [42]. Therefore, our results support that the use of CDF and ACDF containing high Mg concentrations may have possible beneficial effects by reducing oxidative lipid damage during the HD process [11,12,20]. 

Our study has several limitations. First, our results are based on in vitro experiments using a human monocyte cell line. Therefore, experimental results must be viewed with caution and conclusions not inappropriately extrapolated. Secondly, our results might have been affected by other components in DFs, such as some electrolytes present in small concentrations [30]. Therefore, further preclinical and clinical studies will be required to validate our findings. However, scope to measure a wide variety of oxidative-inflammatory stress and damage parameters in different leukocyte populations or other type of cells and tissues (kidney, endothelial cells, etc.) in vivo is very limited, due to (1) limited availability of clinical samples; (2) the wide variety of CKD patient responses to HD therapy; (3) the large variability in Mg serum levels in HD patients, which depends on several factors (dialysate Mg concentration, nutrition, medications, etc.) [26]; (4) the difficulty of patient selection due to the etiology of CKD of the HD patients included in clinical trials sometimes is not representative of the population on dialysis. Nevertheless, this in vivo study is an important starting point, providing useful information about the beneficial effects of high Mg concentration in CDF by reducing oxidative stress and damage in monocytes.

## 5. Conclusions

To the best of our knowledge, our study demonstrates, for the first time, that a high Mg concentration in CDF has beneficial effects on reducing oxidative stress and damage in IS-stimulated THP-1 cells. Although the present study is limited to in vitro experiments, and further confirmatory studies are needed, it shows that an increase of Mg concentration in dialysates may provide a potential therapeutic tool to improve clinical outcomes in CKD patients undergoing HD treatment.

## Figures and Tables

**Table 1:** Composition of the control solution (CS) and the dialysis fluids (DFs) used.

CS or DF Reference	Hank’s Medium	Acetate SoftPac^®^ Baxter	Citrate SelectBag Citrate^®^ Baxter	Acetate + Citrate Citrasate^®^ Nipro
Na, mM	140	140	140	140
K, mM	2	2	2	2
Ca, mM	1.50	1.50	1.65	1.50
Mg, mM	0.50	0.50	0.50	0.50
Cl, mM	106	109.5	106.5	105.7
Citrate, mM	0	0	1	0.8
Acetate, mM	0	3	0	0.3
Bicarbonate, mM	34	34	34	34
Glucose, mg/dL	100	100	100	100

Magnesium sulfate (150 mg/mL solution of MgSO_4_ heptahydrate (Genfarma^®^, Altan Pharma, Madrid, Spain) was used for supplementing the three different DFs (ADF, ACDF, and CDF) and the control solution with Mg concentrations of 1, 1.25, and 2 mM.

## Supplementary Materials

The following are available online at https://www.mdpi.com/2076-3921/9/4/319/s1, supplementary figures: Figure S1; Figure S2; Figure S3. Figure S1: Intracellular reactive oxygen species (ROS) production under basal conditions (control) and in response to indoxyl sulfate (IS; 256 µg/mL) in human THP-1 monocytes cultured in a control solution ((A) Hank’s medium) or various dialysis fluids, (B) ADF (acetate 3 mM), (C) ACDF (citrate 0.3 mM + acetate 0.8 mM), and (D) CDF (citrate 1 mM). Each point represents the mean ± standard deviation (SD) of 8–10 independent experiments. ROS were measured at different times (30, 60, 90, 120, 150, and 180 min), and each value is the mean of duplicate assays. * *p* < 0.05 and ** *p* < 0.01 vs. the value in the basal conditions; Figure S2: Intracellular reactive oxygen species (ROS) production in (A) basal conditions (control) and (B) in response of indoxyl sulfate (IS; 256 µg/mL) in human THP-1 monocytes cultured in Hank’s medium (control solution) with standard Mg (0.5 mM, control) or with high Mg concentrations (1, 1.25, and 2 mM). Each point represents the mean ± SD of 8–10 independent experiments. ROS were measured at different times (30, 60, 90, 120, 150, and 180 min), and each value is the mean of duplicate assays; Figure S3: Intracellular reactive oxygen species (ROS) production under basal conditions (control) and in response of indoxyl sulfate (IS; 256 µg/mL) in human THP-1 monocytes cultured in (A) ADF (acetate 3 mM), (B) ACDF (citrate 0.3 mM + acetate 0.8 mM), and (C) CDF (citrate 1 mM) dialysates with standard Mg (0.5 mM, control) or with high Mg concentrations (1, 1.25, and 2 mM). Each point represents the mean ± SD of 8–10 independent experiments. ROS were measured at different times (30, 60, 90, 120, 150, and 180 min), and each value is the mean of duplicate assays.
