# Supplementary material for: Increasing the Magnesium Concentration in Various Dialysate Solutions Differentially Modulates Oxidative Stress in a Human Monocyte Cell Line
Source: Antioxidants (Basel). 2020 Apr 15;9(4):319. doi: 10.3390/antiox9040319 (PMC7222382; doi:10.3390/antiox9040319)
Supplement: Supplementary file 1 [file antioxidants-09-00319-s001.pdf]

antioxidants

Article

Increasing the Magnesium Concentration in Various Dialysate Solutions Differentially Modulates Oxidative Stress in a Human Monocyte Cell Line

Carmen Vida [1], Julia Carracedo [2,3,*], Patricia de Sequera [4,5], Guillermo Bodega [6], Rafael Pérez [4], Matilde Alique [1,7,*,†] and Rafael Ramírez [1,7,†]

[1] Dpto. de Biología de Sistemas, Universidad de Alcalá, 28871 Alcalá de Henares, Madrid, Spain; carmen.vida@uah.es (C.A.); manuel.ramirez@uah.es (R.R.)
[2] Dpto. Genética, Fisiología y Microbiología (Sección Fisiología), Universidad Complutense de Madrid, 28040 Madrid, Spain
[3] Instituto de Investigación Sanitaria Hospital 12 de Octubre (imas12), 28041 Madrid, Spain
[4] Sección de Nefrología, Hospital Universitario Infanta Leonor, 28031 Madrid, Spain; patricia.desequera@salud.madrid.org (P.d.S.); rperezgarcia@senefro.org (R.P.)
[5] Dpto. de Medicina, Universidad Complutense de Madrid, 2804 Madrid, Spain
[6] Dpto. de Biomedicina y Biotecnología, Universidad de Alcalá, Alcalá de Henares, 28805 Madrid, Spain; guillermo.bodega@uah.es
[7] Instituto Ramón y Cajal de Investigación Sanitaria, (IRYCIS), 28034 Madrid, Spain
* Correspondence: julcar01@ucm.es (J.C.); matilde.alique@uah.es (M.A.)
† These authors share senior authorship

Received: 27 February 2020; Accepted: 14 April 2020; Published: date

Supplementary Materials: The following are available online at www.mdpi.com/xxx/s1, supplementary figures: Figure S1; Figure S2; Figure S3.

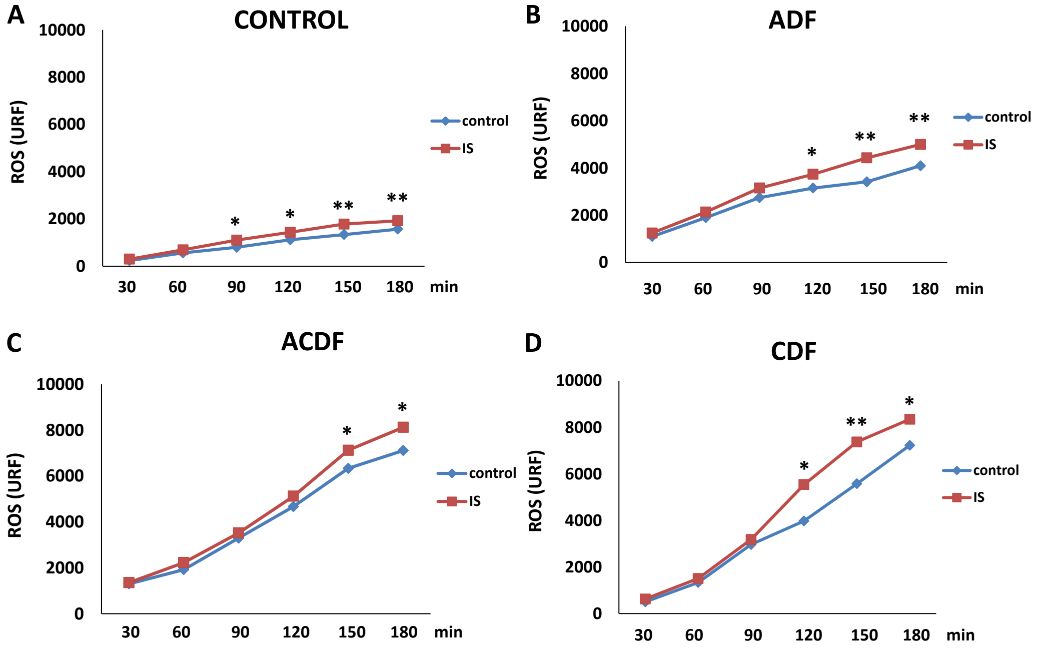

Figure S1. Intracellular reactive oxygen species (ROS) production under basal conditions (control) and in response to indoxyl sulfate (IS; 256 μg/mL) in human THP-1 monocytes cultured in a control solution ((**A**) Hank´s medium) or various dialysis fluids ((B) ADF (acetate 3 mM), (C) ACDF (citrate 0.3 mM + acetate 0.8 mM), and (D) CDF (citrate 1 mM). Each point represents the mean ± standard deviation (SD) of 8–10 independent experiments. ROS were measured at different times (30, 60, 90,

120, 150, and 180 min), and each value is the mean of duplicate assays. * $p < 0.05$ and ** $p < 0.01$ vs. the value in the basal conditions.

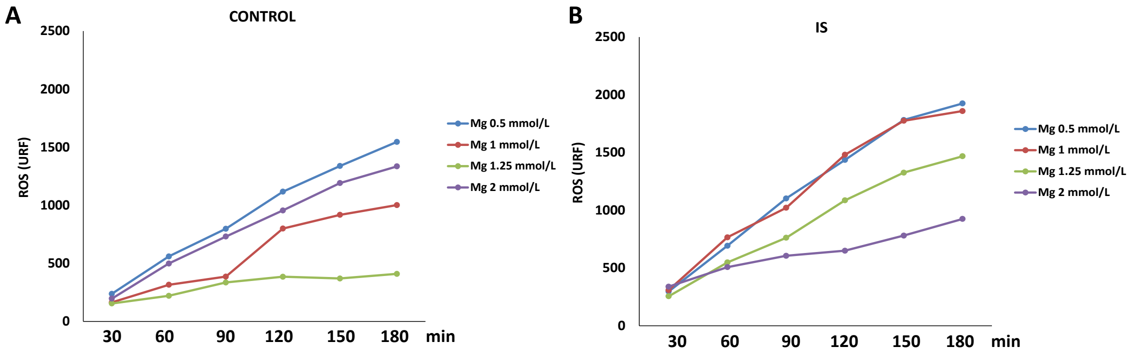

Figure S2. Intracellular reactive oxygen species (ROS) production in (A) basal conditions (control) and (B) in response of indoxyl sulfate (IS; 256 μg/mL) in human THP-1 monocytes cultured in Hank´s medium (control solution) with standard Mg (0.5 mM, control) or with high Mg concentrations (1, 1.25, and 2 mM). Each point represents the mean ± SD of 8–10 independent experiments. ROS were measured at different times (30, 60, 90, 120, 150, and 180 min), and each value is the mean of duplicate assays.

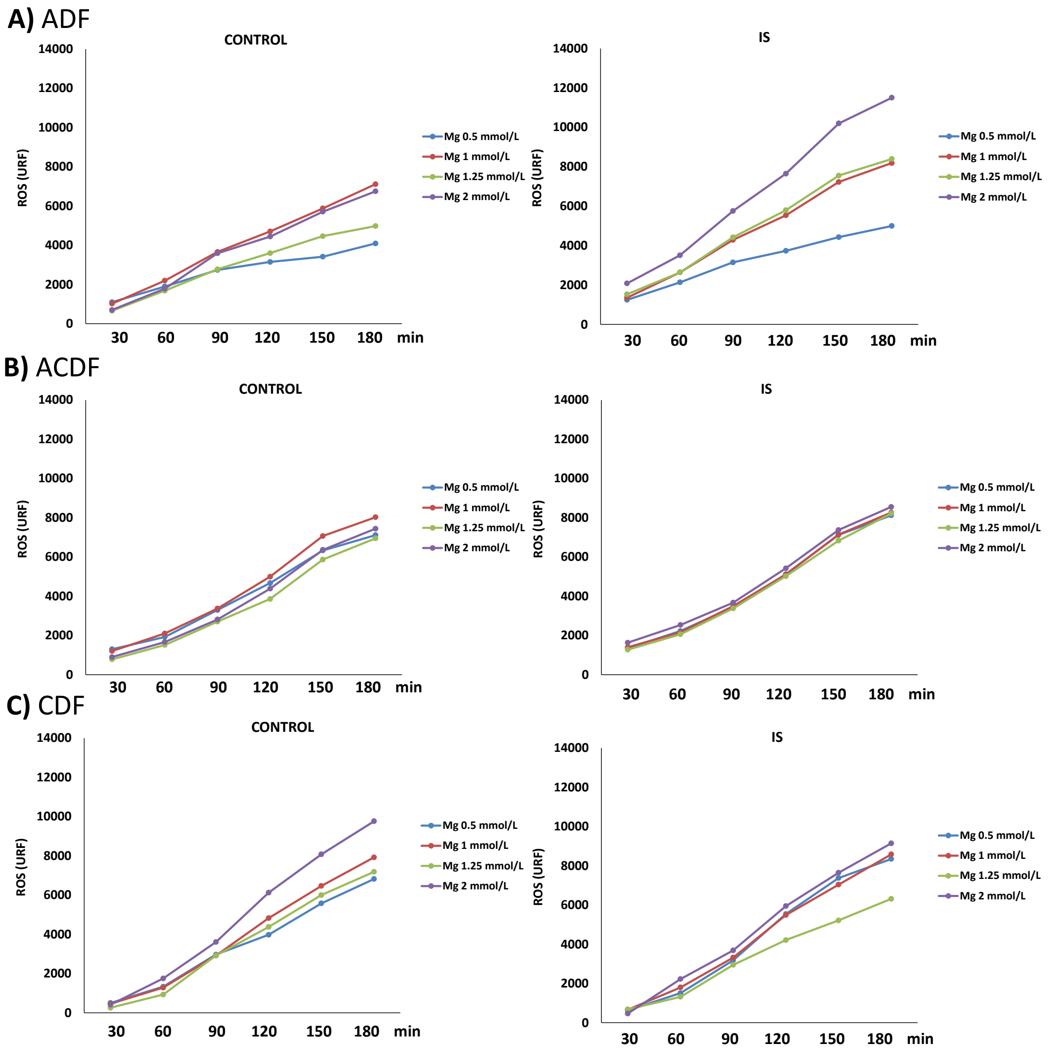

Figure S3. Intracellular reactive oxygen species (ROS) production under basal conditions (control) and in response of indoxyl sulfate (IS; 256 μg/mL) in human THP-1 monocytes cultured in (A) ADF

(acetate 3 mM), (B) ACDF (citrate 0.3 mM + acetate 0.8 mM), and (C) CDF (citrate 1 mM) dialysates with standard Mg (0.5 mM, control) or with high Mg concentrations (1, 1.25, and 2 mM). Each point represents the mean ± SD of 8–10 independent experiments. ROS were measured at different times (30, 60, 90, 120, 150, and 180 min), and each value is the mean of duplicate assays.

© 2020 by the authors. Submitted for possible open access publication under the terms and conditions of the Creative Commons Attribution (CC BY) license (http://creativecommons.org/licenses/by/4.0/).
